# Stability of Rosmarinic Acid and Flavonoid Glycosides in Liquid Forms of Herbal Medicinal Products—A Preliminary Study

**DOI:** 10.3390/ph14111139

**Published:** 2021-11-10

**Authors:** Agnieszka Bodalska, Adam Kowalczyk, Izabela Fecka

**Affiliations:** Department of Pharmacognosy and Herbal Medicines, Faculty of Pharmacy, Wroclaw Medical University, ul. Borowska 211, 50-556 Wroclaw, Poland; adam.kowalczyk@umw.edu.pl (A.K.); izabela.fecka@umw.edu.pl (I.F.)

**Keywords:** herbal medicinal product stability, rosmarinic acid (CAS No: 20283-92-5), luteolin-7-*O*-β-glucuronide (CAS No: 29741-10-4), luteolin-7-*O*-β-glucuronoside, eriocitrin (CAS No: 13463-28-0), eriodictyol-7-*O*-rutinoside

## Abstract

Peppermint leaf, sage leaf, thyme herb, and their preparations are common components of herbal medicinal products (HMPs). According to the European Pharmacopoeia guidelines, the above-mentioned plant substances are standardized for the content of essential oils, omitting polyphenols, which also have a significant impact on their activities. The aim of this study was to evaluate the stability of the predominant polyphenols—rosmarinic acid, luteolin-7-*O*-β-glucuronide, and eriocitrin—in selected commercial liquid HMPs containing thyme, sage, and peppermint under long-term, intermediate, and accelerated testing conditions. Qualitative and quantitative analyses of these polyphenols were performed by the previously optimized and validated HPLC-DAD method. Rosmarinic acid stability was better in hydroethanolic than in an aqueous solution. The effect of the solvent on the stability of luteolin-7-*O*-β-glucuronide and eriocitrin could not be determined and requires further investigation. The present study is the first to analyze the stability of these compounds in commercial herbal medicinal products. The expiration dates proposed by the manufacturers of the tested HMPs did not guarantee stable levels of all analyzed polyphenols throughout the stated period. However, this study is preliminary and requires continuation on a larger number of medicinal products.

## 1. Introduction

Herbal medicinal products (HMPs) are medicines that contain herbal substances or herbal preparations as the only active ingredients [1]. HMPs must fulfill restrictions regarding their characteristics, identification, and purity. An important part of the assessment of the quality of medicinal products, including herbal medicinal products, is the evaluation of their stability. Stability tests allow for the determination of the effects of environmental factors (e.g., temperature, humidity) on active ingredients and thus on a medicine’s quality, efficacy, and safety. The stability of dry herbs depends on the degree of their fragmentation and storage conditions; in airtight conditions, it is about a year [2]. Dried herbal materials—peppermint leaf, sage leaf, and thyme herb—as well as most of their preparations, under the guidelines of the European Pharmacopoeia (Ph. Eur), are standardized for the content of essential oils, with complete disregard for polyphenols, such as phenolic acids and flavonoids, no less relevant for their therapeutic effect [3]. These herbs and their preparations are indicated in the case of dyspepsia (peppermint tincture) and for conditions of mouth, throat, and upper respiratory tract inflammation (thyme and sage preparations) [4]. Rosmarinic acid is widely found in Lamiaceae plants. The beneficial properties of this compound in the treatment of inflammatory diseases (e.g., inflammatory bowel disease, allergic rhinitis, periodontal diseases) by the regulation of inflammatory mediators have been proven [5,6]. Flavonoids such as luteolin, eriodictyol, and their glycosides (e.g., luteolin-7-*O*-β-glucuronide = luteolin-7-*O*-β-glucuronoside and eriocitrin = eriodictyol-7-*O*-rutinoside) show a broad spectrum of activity; both compounds exhibit anti-inflammatory effects [7,8]. Luteolin also exhibits antimicrobial, antiviral, and antispasmodic properties [9,10,11]. Detailed studies on the mechanism of luteolin action in influenza A virus infection have shown that this compound inhibits the formation of coat protein I [10]. A comprehensive review by Aziz and co-workers [7] describes the mechanisms of its anti-inflammatory action, including the inhibition of transcription factors such as NF-κB, NO production, and the inhibition of pro-inflammatory interleukins. It is worth noting that luteolin is metabolized in the human body to glucuronide conjugates (e.g., luteolin-7-*O*-β-glucuronide) [12]. The mechanism of the anti-inflammatory action of luteolin-7-*O*-β-glucuronide was also investigated. As shown, it includes an inhibitory effect on the production of pro-inflammatory factors [13]. The activity profile of eriocitrin, the main peppermint flavonoid, includes lipid-lowering and supportive effects in metabolic diseases [14,15]. In light of the studies cited, it becomes clear that these polyphenol compounds have an essential role in the therapeutic action of the above-mentioned herbs and their pharmaceutical preparations.

The stability of phenolic acids and flavonoid glycosides in liquid medicines has not yet been studied under long-term, intermediate, and accelerated test conditions. The literature reports mainly focus on investigating the stability of the standard solutions of the selected polyphenol compounds under analysis conditions, rather than their stability in liquid HMPs. Therefore, the aim of this study was to evaluate the stability of rosmarinic acid and predominant flavonoids in the selected liquid HMPs based on thyme, sage, and peppermint. The need to standardize the content of polyphenol compounds in herbal substances and preparations of the Lamiaceae family (in addition to essential oil) has been noted by other researchers. This study is the first to analyze the stability of polyphenol compounds in finished HMPs.

## 2. Results and Discussion

**Figure 1:**
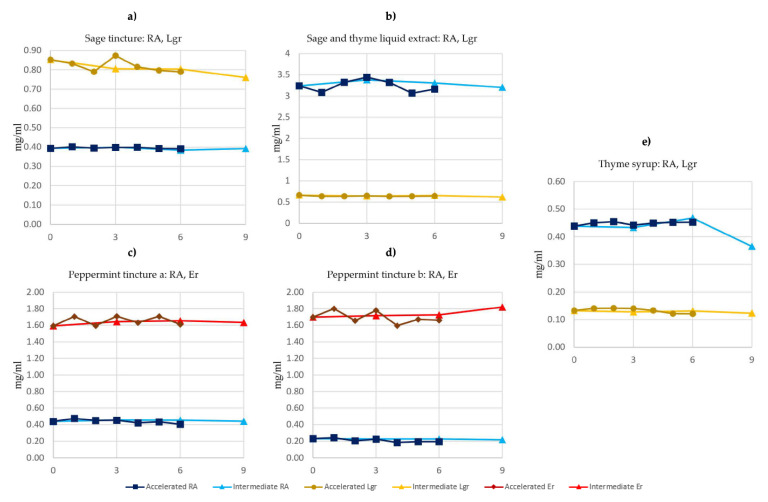
Rosmarinic acid (RA), luteolin-7-*O*-β-glucuronide (Lgr), and eriocitrin (Er) content in accelerated and intermediate tests of: (**a**) Sage tincture; (**b**) sage and thyme liquid extract; (**c**) peppermint tincture a; (**d**) peppermint tincture b; (**e**) thyme syrup.

During the experiment, the stability of rosmarinic acid and the predominant flavonoid compounds in five batches of liquid HMPs was analyzed under three different storage conditions—accelerated, intermediate, and long-term study. Both the conditions of the tests and their duration were under the European Medicines Agency (EMA) and the International Council for Harmonisation of technical requirements for pharmaceuticals for human use (ICH) guidelines. Figure 1 presents the degradation profiles of analyzed polyphenols in the accelerated and intermediate study. The degradation kinetics of many therapeutic substances are consistent with first-order kinetics. However, to provide a more readable presentation of the results, the data obtained under long-term test conditions were presented in logarithmic form (ln % content). These results were used to estimate the HMPs’ shelf life. Figures S1–S5 in supplementary data present HPLC chromatograms of analyzed products at the three measurement points: 0, 9th (accelerated test conditions), and 24th month (long-term test conditions).

### 2.1. Sage Tincture (ST)

No significant change in rosmarinic acid content was observed when the tincture was stored under accelerated and intermediate conditions. The initial concentration was 0.39 mg/mL. The concentration variations were in the range of 99.4–102.1% of the initial content for the accelerated test and 97.5–101.4% for the intermediate test. Fluctuations in the luteolin-7-*O*-β-glucuronide concentration were more pronounced (Figure 1a). In the accelerated test, from an initial concentration of 0.85 mg/mL, there was a decrease to 0.79 mg/mL (92.7%), while in the intermediate test, there was a decrease to 0.76 mg/mL (89.2%).

**Figure 2:**
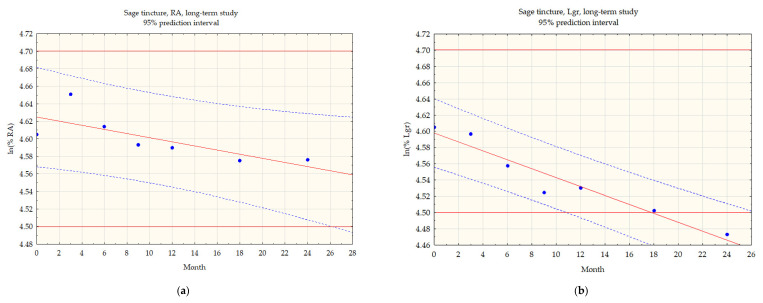
Sage tincture, (**a**) rosmarinic acid (RA) content in long-term test, (**b**) luteolin-7-*O*-β-glucuronide (Lgr) content in long-term test.

The stability of rosmarinic acid was demonstrated over the tested time interval during the long-term test. At 24 months, the concentration of rosmarinic acid decreased to 97.1% of the initial value and was 0.37 mg/mL. Based on the concentration of rosmarinic acid, the estimated shelf life of the sage tincture is about 26 months (Figure 2a). The second compound analyzed, luteolin-7-*O*-β-glucuronide, was found to be less stable. Its concentration dropped below 95% as early as month 9 of the study, and at the last measurement point was 0.74 mg/mL (87.6%). The estimated period that luteolin-7-*O*-β-glucuronide content remains above 90% is about 11 months (Figure 2b). Numeric data for sage tincture samples are presented in Table S1.

### 2.2. Sage and Thyme Liquid Extract (STE)

The initial content of rosmarinic acid in STE samples was 3.24 mg/mL. During the accelerated study, fluctuations in the concentration ranging from 94.6 to 102.5% were observed; after 6 months, this value was 3.16 mg/mL (97.6%). For the intermediate study, the level of rosmarinic acid was relatively constant, reaching 3.20 mg/mL (98.9%) at the end of the test. The concentration of luteolin-7-*O*-β-glucuronide also remained stable, falling from an initial value of 0.66 mg/mL to 0.64 mg/mL in the accelerated study, while in the intermediate study, a visible decrease was observed only at the last measurement point after 9 months—0.62 mg/mL (93.3%). The abovementioned data are illustrated in Figure 1b.

The stability of rosmarinic acid in STE, estimated in the long-term test, is 22 months. During the first year and a half of the study, fluctuations in concentration did not exceed ± 5% of the initial content until month 24, when it dropped to 94.8% (3.07 mg/mL). Luteolin-7-*O*-β-glucuronide showed greater stability; throughout the long-term study its concentration did not drop below 95% (0.63 mg/mL at the last measurement point). The estimated time for the luteolin-7-*O*-β-glucuronide concentration not decreasing below 90% of the initial value is 28 months. Figures S6 and S7 present the scatterplots with 95% confidence intervals of concentrations of these two polyphenols, while the numerical data regarding STE samples are shown in Table S2.

### 2.3. Peppermint Tincture (PT)

Two different batches of peppermint tincture (PTa and PTb) were subjected to analysis. These batches differed not only in their rosmarinic acid stability but also in their initial concentration, which was 0.44 mg/mL for the PTa batch and 0.23 mg/mL for PTb. In the accelerated test for the PTa batch, the concentration did not fall below 95% for the first 5 months, but was 0.41 mg/mL (92.0%) at the last measurement point. Rosmarinic acid in the intermediate test was stable in the range 100–103.3% (0.44–0.45 mg/mL). For the PTb batch, there was a large decrease in the rosmarinic acid content in the accelerated test, where a concentration of 0.18 mg/mL (79.6%) was observed at month 4 of the study, then for two consecutive months, the content remained at 0.19 mg/mL (83.9%). In the intermediate test, the content gradually decreased to reach 0.22 mg/mL (93.8%) at the end of the study.

The initial concentrations of the second compound tested, eriocitrin, were 1.59 mg/mL for PTa and 1.70 mg/mL for PTb. Both peppermint tinctures showed stability of this compound in the accelerated study; its concentration ranged between 1.59 and 1.71 mg/mL (100–107.2%) for PTa and 1.80–1.60 mg/mL (106.0–93.9%) for PTb. Eriocitrin stability was also observed during the intermediate study: 1.59–1.65 mg/mL (100–103.8%) for PTa and 1.70–1.82 mg/mL (100–107.1%) for PTb.

Analysis of the long-term test results allowed us to estimate the shelf life of the tested tinctures. In the case of rosmarinic acid in sample PTa, this period is 10 months (Figure S8). The concentration of this compound from the initial 0.44 mg/mL increased in the second month to 0.47 mg/mL and gradually decreased to reach 0.40 mg/mL (90.9%) at the end of the test. For the PTb batch, the estimated shelf life is approximately 21 months (Figure S10). The rosmarinic acid concentration after two years of the study was 0.22 mg/mL (94.2%).

Furthermore, the analysis of changes in eriocitrin content produced different results for the tested batches. For PTa, the content of this flavonoid increased and remained at 106.3–107% at months 3, 6, and 9 of the study and then began to decrease. For the PTb batch, concentration changes were observed in the range of 1.77–1.60 mg/mL (105.7–94.1%). Again, this batch was shown to have higher stability of 16 months (Figure S11), while for PTa this period was estimated at 10 months (Figure S9). Figure 1c,d, and Tables S3 and S4 present data regarding PTa and PTb.

The slight increase observed in rosmarinic acid and eriocitrin content during the storage of peppermint tinctures may be related to the volatilization of small solvent volumes (ethanol). On the other hand, it is within the statistical error (Tables S3 and S4).

### 2.4. Thyme Syrup (TS)

The initial content of rosmarinic acid in thyme syrup was 0.44 mg/mL. In the accelerated study, the concentration range was 100–103.7%. Larger variations in the content of this compound were observed during the intermediate study, at 6 and 9 months—0.47 mg/mL (106.7%) and 0.36 mg/mL (83.3%), respectively. The luteolin-7-*O*-β-glucuronide concentration decreased from an initial 0.13 mg/mL to 0.12 mg/mL during both the accelerated test and the long-term test.

In the long-term study, rosmarinic acid was stable until the 18th month, with a content of 0.44 mg/mL 102%, then at the last measurement point its concentration dropped significantly to 0.25 mg/mL (54.6%). Such concentration changes over time did not correspond to zero- or first-order degradation kinetics (Figure S12). Variations in content were also observed for luteolin-7-*O*-β-glucuronide. During the third and sixth months, the concentration remained at 0.14 mg/mL (106%) and then began to decrease. At the last measurement point, it reached 0.12 mg/mL (92%). According to the estimation, the stability of luteolin-7-*O*-β-glucuronide in thyme syrup is about 14 months (Figure S13). Data regarding thyme syrup samples are presented in Figure 1d and Table S5.

### 2.5. Stability of Polyphenols and HMP Expiration Dates

**Figure 3:**
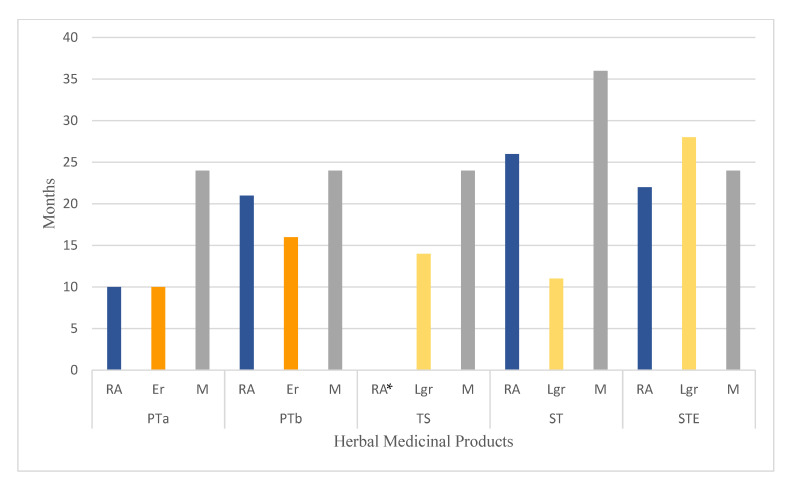
Determined expiration dates of the analyzed HMPs based on the stability of rosmarinic acid and flavonoid glycosides. RA—rosmarinic acid, Er—eriocitrin, Lgr—luteolin-7-*O*-β-glucuronide, M—manufacturer’s shelf-life; PTa, PTb—peppermint tinctures, batches a and b; TS—thyme syrup, ST—sage tincture, STE—sage and thyme liquid extract; RA*—stability of rosmarinic acid for thyme syrup could not be estimated.

This study allowed us to estimate the expiration date of the analyzed liquid herbal medicines considering the stability of polyphenols. The results were compared with the term stated by the manufacturer and are presented in Figure 3. The product with the highest stability of rosmarinic acid (26 months) and the lowest stability of luteolin-7-*O*-β-glucuronide (11 months) was sage tincture. According to the manufacturer’s guidelines, its expiration period is three years. This means that a drug administered after more than two years contains a significantly reduced content of flavonoids that may affect the product’s effectiveness. Indications for the use of sage tincture are inflammation of the gums, mouth, and throat. The study conducted by Martins et al. [16] indicated that rosmarinic acid and luteolin-7-*O*-β-glucuronide exhibit antifungal activity against *Candida* sp. Other studies have shown the anti-inflammatory effects of luteolin-7-*O*-β-glucuronide by downregulating nitric oxide, IL-6, and TNF-α synthesis [17]. In their study, Walch and co-workers [18] found a correlation between the sum of rosmarinic acid derivatives in sage infusions and the levels obtained in the oxygen radical absorbance capacity test, as well as the Folin-–Ciocalteu index, and noted the lack of regulation in pharmacopeial guidelines regarding the content of polyphenol compounds in preparations from this plant.

The estimated stability of luteolin-7-*O*-β-glucuronide (28 months) in the sage and thyme liquid extract for external use (STE) even exceeded the manufacturer’s indication (24 months), while for rosmarinic acid, the period was 22 months, so it can be assumed that a stable concentration of polyphenols is ensured during the entire period of use.

The spasmolytic effect of thyme extracts is beneficial in the treatment of bronchitis or respiratory tract infections. This action, according to studies, is not only due to the presence of thymol and carvacrol, but also to the polyphenolic fraction, especially luteolin [11]. Moreover, thyme extract has a relaxant effect on the trachea by interacting with endothelin receptors [19]. An aqueous extract of *T. vulgaris* had a significant impact on the growth of *Streptococcus mutans*, *S. epidermidis*, *Escherichia coli*, *Pseudomonas aeruginosa*, and *Proteus vulgaris*. In comparison, the hydroalcoholic extract had the strongest antibacterial activity against *E. coli* and *P. vulgaris* strains [20]. Researchers have also noted the antimicrobial activity of thyme extracts, which may have applications in dentistry and oral infections [21,22]. The mentioned research provides rationale for drawing more attention to the content of polyphenols in the analyzed HMPs.

The product with the shortest estimated shelf life was peppermint tincture (RA, Er: 10 months). However, it should be emphasized that during the study, the shelf life of the product PTa was exceeded before the sixth measurement point (18 months). In the case of the second batch, the expiration date was exceeded only before the last measurement point. The estimated stability of PTb is 21 months considering the data for rosmarinic acid and 16 months for eriocitrin. The manufacturer’s assigned expiration date—24 months—is close to that estimated from measuring rosmarinic acid concentrations. It is important to note that the analysis included commercial products purchased in a pharmacy, so it was not possible to examine them on the manufacturing date. However, all the HMPs selected were of the most recent production date and the longest time until the expiration. According to Ph. Eur., one of the peppermint preparations—dry extract—is standardized for the content of rosmarinic acid, but the determination of this compound is not a part of the quality testing of peppermint tincture. In his work, Olennikov [23] emphasizes the need to verify the polyphenol content to ensure the quality of *Menthae piperitae folium*. Peppermint’s main flavonoid, eriocitrin, lowers serum VLDL, LDL, and total cholesterol levels, and increases bile secretion. It also prevents insulin resistance, so it may be a valuable compound in the treatment of metabolic diseases associated with a high-fat diet and obesity [24,25].

Due to the non-specific degradation kinetics of rosmarinic acid in thyme syrup, it was not possible to estimate its stability in this preparation. Until the 18th month of the test, its content remained high, but after 24 months it decreased drastically. Again, it is important to emphasize that the measurements at months 18 and 24 of the study were performed after the expiration date. The addition of ammonia to the formulation may also contribute to this process of decomposition. The negative effect of alkaline pH on the stability of polyphenols was reported by Friedman et al. [26]. Higher pH also decreases the antioxidant properties of plant extracts [27]. Other factors affecting the stability of phenolic acids are the type of solvent, storage temperature, and light. According to Razboršek [28], higher temperature accelerates the degradation of compounds. In a study by Zhang and co-workers [29], over the experiment’s duration (13 days), no significant changes in rosmarinic acid concentrations were found across the range of temperatures and light conditions tested (−10 °C, 4 °C, room temperature, 40 °C; all temperatures with and without light). However, as reported by Razboršek [28], higher temperature and light as well as a protic solvent (such as ethanol, methanol, or water) can lead to the isomerization of trans-rosmarinic acid, which occurs naturally in plants, to cis-rosmarinic acid, which does not occur in nature. This study also showed that rosmarinic acid in a solid state was stable throughout the study period (6 months) [28].

To summarize, the herbal medicines tested generally showed low polyphenol content expressed per milliliter of the product. Rosmarinic acid was more stable in a hydroethanolic solution than in an aqueous solution under conditions of stability tests. However, in thyme syrup, its decomposition curve followed a non-specific path, making it impossible to assess its durability. It was probably affected by the addition of ammonia and the change in pH of the extraction mixture. This study did not determine the effect of the solvent on the stability of luteolin-7-*O*-β-glucuronide, as both the highest (sage and thyme liquid extract, STE) and lowest (sage tincture, ST) stability formulations of the compound were hydroethanolic. The concentration of eriocitrin was tested in only one type of HMP, peppermint tincture (hydroethanolic solution), so it was not possible to determine the effect of the solvent on the stability of this flavonoid.

## 3. Materials and Methods

### 3.1. Solvents and Chemicals

All reagents used in this study were of analytical grade. Acetonitrile, methanol, and 90–100% formic acid, purchased from Sigma Aldrich (St. Louis, MO, USA), were of HPLC gradient grade. Water was obtained in the process of distillation and deionization with a Hydrolab Deionizer HLP20UV(Hydrolab, Straszyn, Poland).

### 3.2. Reference Compounds and Standard Solutions

Three reference compounds were used in this study: Rosmarinic acid, RA (Extrasynthese Genay, France; HPLC purity > 99%); luteolin-7-*O*-β-glucuronide = luteolin-7-*O*-β-glucuronoside, Lgr (isolated from *Serpylli herba*; HPLC-DAD purity > 95%); eriocitrin = eriodictyol-7-*O*-rutinoside, Er (isolated from *Menthae piperitae folium*, HPLC-DAD purity > 95%) [30,31]. The preparation of standard solutions and validation parameters (linearity, coefficients of determination, linear ranges, limits of detection (LOD) and quantitation (LOQ)) of authentic standards for the HPLC-DAD method can be found in previous research [32].

### 3.3. HMPs and Sample Preparation

Four types of HMPs containing thyme, sage, and mint extracts were purchased from local pharmacies in 2017. Table 1 presents summarized data of selected HMPs. At the start of the study, all the HMPs analyzed were at the beginning of their shelf life. Two medicines for internal use were examined—peppermint tincture (two different batches, PTa and PTb) and thyme syrup (TS)—and two for external use: Sage tincture (ST) and a herbal medicinal product containing sage and thyme liquid extracts (STE). These medicines were produced by domestic manufacturers: Hasco-Lek (Wrocław, Poland), Herbapol (Kraków, Poland), and Phytopharm (Klęka, Poland). TS was diluted with water in a 1:5 ratio; the other products were diluted with 80% methanol in a 1:20 ratio (ST, STE) or with 50% methanol in a 1:5 ratio (PT). The resulting solutions were filtered through a 0.45 µm Durapore filter (Millipore, Burlington, MA, USA) and subjected to quantitative and qualitative HPLC-DAD analysis.

### 3.4. HPLC-DAD Conditions and Quantification

The HPLC-DAD analysis conditions were set as in a previous paper [32]. The method has undergone validation according to ICH guidelines Q2 Analytical Validation [33]. Values were calculated from 3 independent samples and measurements. Results are given as the mean with the %CV (coefficient of variation) range.

### 3.5. Stability Tests and Data Analysis

The analyzed HMPs were stored in their original packaging under the conditions of three stability tests: Accelerated, intermediate, and long-term. The minimum time period for those studies is six months. The accelerated test was conducted in a Binder KBF LQC-240 climate chamber (Binder, Germany), 40 ± 2 °C/75 ± 5% relative humidity (RH); the samples were tested for six months, at monthly intervals. The intermediate test was carried out in a Binder KBW 720 climate chamber (Binder, Germany), at 30 ± 2 °C and 65 ± 5% RH. Polyphenol concentration measurement was performed at 0, 3, 6, and 9 months. The long-term test was conducted in a laboratory with a constant temperature of 25 ± 2 °C and 60 ± 5% RH. For the first year, samples were tested every three months, and the next measurement points were after 18, 24, and 36 months. Each measurement was repeated three times. Storage conditions were in accordance with the EMA and ICH guidelines [34,35].

In order to estimate the shelf life of selected HMPs, the data obtained during the long-term test were subjected to statistical analysis. As the degradation of most compounds follows first-order kinetics, polyphenol concentrations were converted to a percentage relative to the initial content and then logarithmized. After the transformation of the experimental data, a scatter plot with a 95% confidence interval was drawn to determine the shelf life. Linear regression analysis was performed along with an F-test of the logarithmized data. The shelf life is estimated based on changes in the content of tested compounds and is defined as the point at which the confidence interval of the scatterplot of the concentration of a given compound crosses 90% of the initial content, assuming a logarithmic dependence of the concentration on time.

## 4. Conclusions

This study provides a broader view of the stability of rosmarinic acid, luteolin-7-*O*-β-glucuronide, and eriocitrin in liquid pharmaceutical formulations of thyme, sage, and peppermint. The research presented here is only preliminary, but it suggests that the stability of polyphenols such as phenolic acids and flavonoid glycosides in liquid preparations varies. We showed that the expiration dates proposed by the manufacturers for the examined HMPs did not guarantee stable levels of all analyzed polyphenols throughout the stated period. Therefore, more extensive research on the stability of phenolic acids and flavonoid glycosides in different formulations of herbal medicines appears to be necessary.

## Figures and Tables

**Table 1:** Basic data on the analyzed herbal medicinal products.

Acronym of the HMP	Form	Ingredients	Indications	Expiration Date (Months)
**Preparations for External Use**				
ST	Sage tincture	Sage leaf tincture (*S. officinalis* L.) DER 1:5	Inflammation of the mouth, gums and throat	36
STE	Liquid spray	Sage leaf tincture (*S. officinalis* L.), Thyme herb liquid extract (*T. vulgaris* L. or *T. zygis* L.),	Inflammation of the mouth and throat	24
**Preparations For Internal Use**				
PT	Peppermint tincture	Peppermint tincture (*M.* x *piperita* L.) DER 1:19.7–21	Mild digestive disorders, dyspepsia, flatulence, intestinal cramps	24
TS	Thyme syrup	Thyme herb liquid extract (*T. vulgaris* or *T. zygis* L.) DER 1:3	Cough with upper respiratory tract rhinitis, difficult expectoration	24

## Data Availability

Data are contained within the article and supplementary material.

## Supplementary Materials

The following are available online: https://www.mdpi.com/article/10.3390/ph14111139/s1, Supplementary data—Tables: Table S1: Concentrations of rosmarinic acid (RA) and luteolin-7-*O*-β-glucuronide (Lgr) in sage tincture (ST) samples in the stability tests; Table S2: Concentrations of rosmarinic acid (RA) and luteolin-7-*O*-β-glucuronide (Lgr) in sage and tincture extract (STE) samples in the stability tests; Table S3: Concentrations of rosmarinic acid (RA) and eriocitrin (Er) in peppermint tincture a (PTa) samples in the stability tests; Table S4: Concentrations of rosmarinic acid (RA) and eriocitrin (Er) in peppermint tincture b (PTb) samples in the stability test; Table S5: Concentrations of rosmarinic acid (RA) and luteolin-7-*O*-β-glucuronide (Lgr) in thyme syrup (TS) samples in the stability tests. Figures: Figure S1: HPLC chromatograms of sage tincture (ST) in the three measurement points: 0, 9th (intermediate test), 24th month (long-term test) at 320 nm; Figure S2: HPLC chromatograms of sage and thyme liquid extract (STE) in the three measurement points: 0, 9th (intermediate test), 24th month (long-term test) at 320 nm; Figure S3: HPLC chromatograms of peppermint tincture a (PTa) in the three measurement points: 0, 9th (intermediate test), 24th month (long-term test) at 320 nm; Figure S4: HPLC chromatograms of peppermint tincture b (PTb) in the three measurement points: 0, 9th (intermediate test), 24th month (long-term test) at 320 nm; Figure S5: HPLC chromatograms of thyme syrup (TS) in the three measurement points: 0, 9th (intermediate test), 24th month (long-term test) at 320 nm; Figure S6: Sage and thyme liquid extract, rosmarinic acid (RA) content in the long-term test.; Figure S7: Sage and thyme liquid extract, luteolin-7-*O*-β-glucuronide (Lgr) content in the long-term test; Figure S8: Peppermint tincture a, rosmarinic acid (RA) content in the long-term test; Figure S9: Peppermint tincture a, eriocitrin (Er) content in the long-term test; Figure S10: Peppermint tincture b, rosmarinic acid (RA) content in the long-term test; Figure S11: Peppermint tincture b, eriocitrin (Er) content in the long-term test; Figure S12: Thyme syrup, rosmarinic acid (RA) content in the long-term test; Figure S13: Thyme syrup, luteolin-7-*O*-β-glucuronide (Lgr) content in the long-term test.
