# Supplementary material for: Stability of Rosmarinic Acid and Flavonoid Glycosides in Liquid Forms of Herbal Medicinal Products—A Preliminary Study
Source: Pharmaceuticals (Basel). 2021 Nov 10;14(11):1139. doi: 10.3390/ph14111139 (PMC8624675; doi:10.3390/ph14111139)
Supplement: Supplementary file 1 [file pharmaceuticals-14-01139-s001.zip › pharmaceuticals-1437481-supplementary_noComments.pdf]

Supplementary data to:

'Stability of rosmarinic acid and flavonoid glycosides in liquid forms of herbal medicinal products – a preliminary study'

Agnieszka Bodalska, Adam Kowalczyk, Izabela Fecka

Department of Pharmacognosy and Herbal Medicines, Faculty of Pharmacy, Wroclaw Medical University, ul. Borowska 211, 50-556 Wroclaw, Poland

List of Tables:

Table S1. Concentrations of rosmarinic acid (RA) and luteolin-7-*O*-β-glucuronide (Lgr) in sage tincture (ST) samples in the stability tests.

Table S2. Concentrations of rosmarinic acid (RA) and luteolin-7-*O*-β-glucuronide (Lgr) in sage and tincture extract (STE) samples in the stability tests.

Table S3. Concentrations of rosmarinic acid (RA) and eriocitrin (Er) in peppermint tincture a (PTa) samples in the stability tests.

Table S4. Concentrations of rosmarinic acid (RA) and eriocitrin (Er) in peppermint tincture b (PTb) samples in the stability tests.

Table S5. Concentrations of rosmarinic acid (RA) and luteolin-7-*O*-β-glucuronide (Lgr) in thyme syrup (TS) samples in the stability tests.

List of Figures:

Figure S1. HPLC chromatograms of sage tincture (ST) in the three measurement points: 0, 9th (intermediate test), 24th month (long-term test) at 320 nm.

Figure S2. HPLC chromatograms of sage and thyme liquid extract (STE) in the three measurement points: 0, 9th (intermediate test), 24th month (long-term test) at 320 nm.

Figure S3. HPLC chromatograms of peppermint tincture a (PTa) in the three measurement points: 0, 9th (intermediate test), 24th month (long-term test) at 320 nm.

Figure S4. HPLC chromatograms of peppermint tincture b (PTb) in the three measurement points: 0, 9th (intermediate test), 24th month (long-term test) at 320 nm.

Figure S5. HPLC chromatograms of thyme syrup (TS) in the three measurement points: 0, 9th (intermediate test), 24th month (long-term test) at 320 nm.

Figure S6. Sage and thyme liquid extract, rosmarinic acid (RA) content in the long-term test.

Figure S7. Sage and thyme liquid extract, luteolin-7-*O*-β-glucuronide (Lgr) content in long-term test.

Figure S8. Peppermint tincture a, rosmarinic acid (RA) content in the long-term test.

Figure S9. Peppermint tincture a, eriocitrin (Er) content in the long-term test.

Figure S10. Peppermint tincture b, rosmarinic acid (RA) content in the long-term test.

Figure S11. Peppermint tincture b, eriocitrin (Er) content in the long-term test.

Figure S12. Thyme syrup, rosmarinic acid (RA) content in the long-term test.

Figure S13. Thyme syrup, luteolin-7-*O*-β-glucuronide (Lgr) content in the long-term test.

Table S1. Concentrations of rosmarinic acid (RA) and luteolin-7-*O*-β-glucuronide (Lgr) in sage tincture (ST) samples in the stability tests.

Accelerated			Intermediate			Long-term		
Month	RA	Lgr	Month	RA	Lgr	Month	RA	Lgr
0	0,39[a]	0,85[a]	0	0,39[a]	0,85[a]	0	0,38[a]	0,85[a]
1	0,40[b]	0,83[a]	3	0,40[b]	0,80[a]	3	0,40[b]	0,84[b]
2	0,39[a]	0,79[c]	6	0,38[a]	0,80[b]	6	0,39[a]	0,81[a]
3	0,40[b]	0,87[a]	9	0,39[b]	0,76[b]	9	0,38[a]	0,78[a]
4	0,40[b]	0,82[b]				12	0,38[a]	0,78[a]
5	0,39[b]	0,80[a]				18	0,37[a]	0,76[b]
6	0,39[a]	0,79[b]				24	0,37[c]	0,74[a]

[a]less than 1% CV, [b]less than 3% CV, [c]less than 5% CV

Table S2. Concentrations of rosmarinic acid (RA) and luteolin-7-*O*-β-glucuronide (Lgr) in sage and tincture extract (STE) samples in the stability tests.

Accelerated			Intermediate			Long-term		
Month	RA	Lgr	Month	RA	Lgr	Month	RA	Lgr
0	3,24[a]	0,66[b]	0	3,24[a]	0,66[b]	0	3,24[a]	0,66[b]
1	3,08[a]	0,64[a]	3	3,38[a]	0,64[a]	3	3,39[b]	0,67[a]
2	3,32[a]	0,63[b]	6	3,31[a]	0,65[a]	6	3,35[a]	0,64[a]
3	3,44[a]	0,65[a]	9	3,20[a]	0,62[b]	9	3,27[a]	0,64[a]
4	3,32[a]	0,63[a]				12	3,20[a]	0,64[a]
5	3,07[a]	0,64[a]				18	3,14[a]	0,64[b]
6	3,16[a]	0,64[a]				24	3,07[a]	0,63[b]

[a]less than 1% CV, [b]less than 3% CV

Table S3. Concentrations of rosmarinic acid (RA) and eriocitrin (Er) in peppermint tincture a (PTa) samples in the stability tests.

Accelerated			Intermediate			Long-term		
Month	RA	Er	Month	RA	Er	Month	RA	Er
0	0,44[c]	1,59[c]	0	0,44[c]	1,59[c]	0	0,44[c]	1,59[c]
1	0,48[a]	1,71[a]	3	0,45[b]	1,64[a]	3	0,47[b]	1,71[a]
2	0,45[c]	1,60[c]	6	0,45[a]	1,65[a]	6	0,47[a]	1,69[a]
3	0,45[a]	1,71[a]	9	0,44[a]	1,63[c]	9	0,47[a]	1,70[b]
4	0,42[a]	1,63[a]				12	0,45[b]	1,55[c]
5	0,43[a]	1,71[a]				18	0,42[a]	1,52[a]
6	0,41[a]	1,61[a]				24	0,40[a]	1,48[c]

[a]less than 1% CV, [b]less than 3% CV, [c]less than 5% CV

Table S4. Concentrations of rosmarinic acid (RA) and eriocitrin (Er) in peppermint tincture b (PTb) samples in the stability tests.

Accelerated			Intermediate			Long-term		
Month	RA	Er	Month	RA	Er	Month	RA	Er
0	0,23[b]	1,70[a]	0	0,23[b]	1,70[a]	0	0,23[b]	1,70[a]
1	0,24[a]	1,80[a]	3	0,23[a]	1,72[a]	3	0,23[a]	1,80[a]
2	0,21[a]	1,66[a]	6	0,23[a]	1,73[a]	6	0,23[a]	1,76[a]
3	0,23[a]	1,78[a]	9	0,22[a]	1,82[a]	9	0,23[a]	1,77[a]
4	0,18[c]	1,60[c]				12	0,22[a]	1,68[a]
5	0,19[c]	1,67[c]				18	0,22[b]	1,62[a]
6	0,19[a]	1,66[a]				24	0,22[a]	1,60[a]

[a]less than 1% CV, [b]less than 3% CV, [c]less than 5% CV

Table S5. Concentrations of rosmarinic acid (RA) and luteolin-7-*O*-β-glucuronide (Lgr) in thyme syrup (TS) samples in the stability tests.

Accelerated			Intermediate			Long-term		
Month	RA	Lgr	Month	RA	Lgr	Month	RA	Lgr
0	0,44[b]	0,13[b]	0	0,44[b]	0,13[b]	0	0,44[b]	0,13[b]
1	0,45[b]	0,14[b]	3	0,43[a]	0,13[c]	3	0,48[b]	0,14[b]
2	0,45[b]	0,14[c]	6	0,47[b]	0,13[c]	6	0,44[b]	0,14[b]
3	0,44[b]	0,14[a]	9	0,37[b]	0,12[a]	9	0,44[b]	0,14[b]
4	0,45[b]	0,13[b]				12	0,46[b]	0,13[b]
5	0,45[a]	0,12[c]				18	0,44[a]	0,13[b]
6	0,45[b]	0,12[b]				24	0,24[c]	0,12[c]

[a]less than 1% CV, [b]less than 3% CV, [c]less than 5% CV

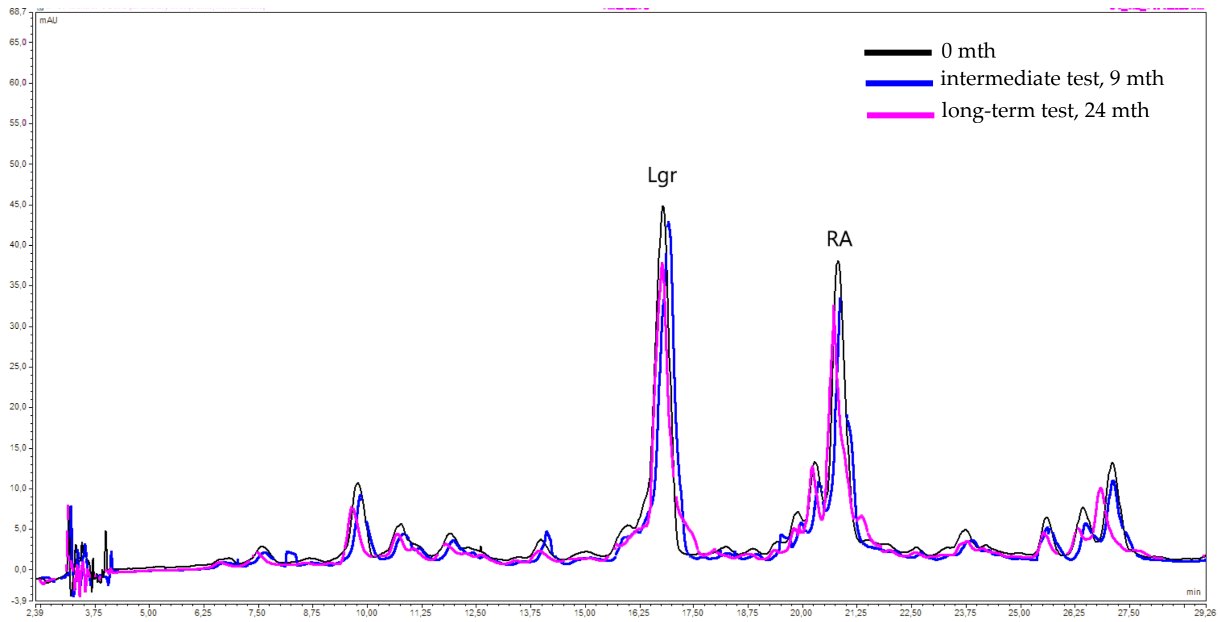

Figure S1. HPLC chromatograms of sage tincture (ST) in the three measurement points: 0, 9th (intermediate test), 24th month (long-term test) at 320 nm.

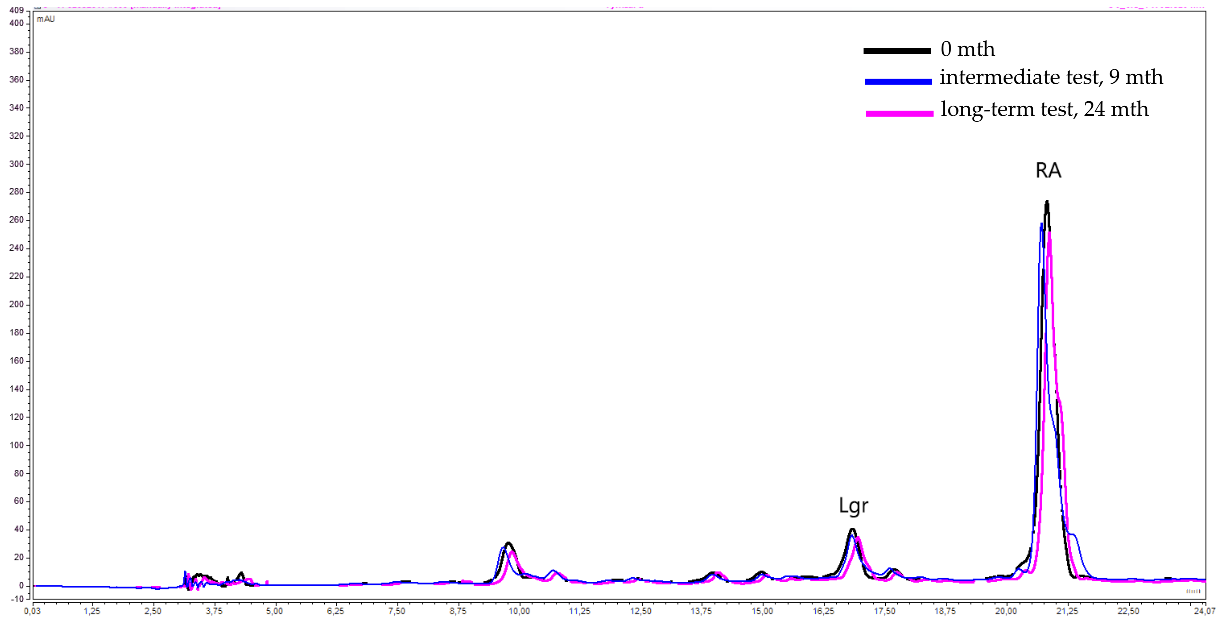

Figure S2. HPLC chromatograms of sage and thyme liquid extract (STE) in the three measurement points: 0, 9th (intermediate test), 24th month (long-term test) at 320 nm.

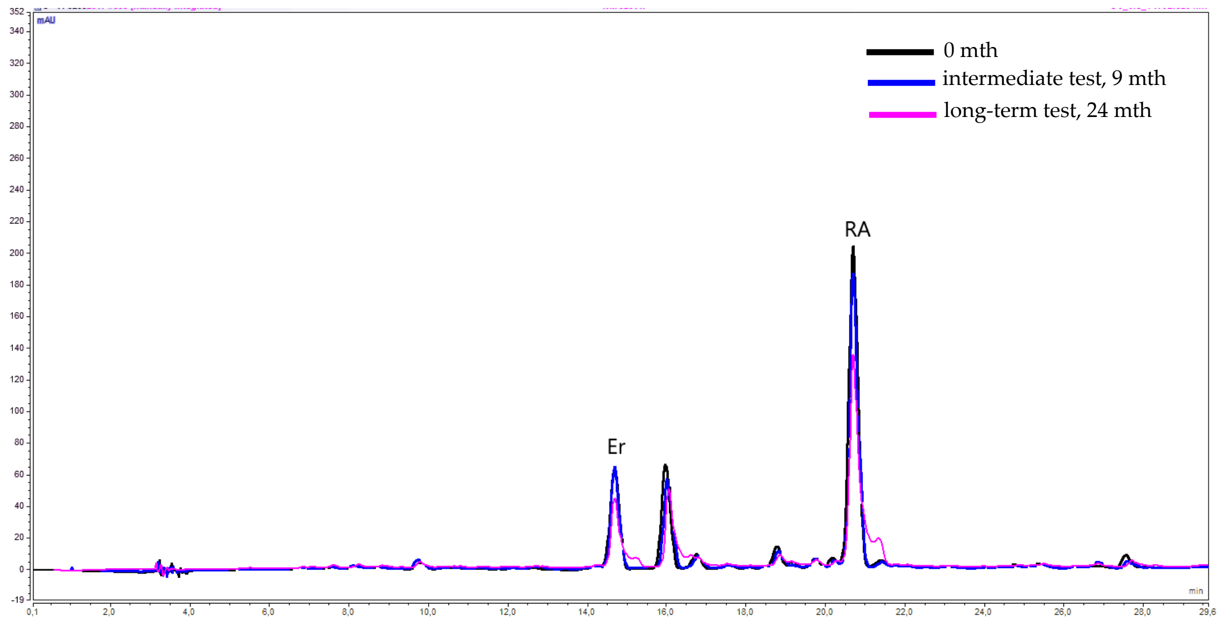

Figure S3. HPLC chromatograms of peppermint tincture a (PTa) in the three measurement points: 0, 9th (intermediate test), 24th month (long-term test) at 320 nm.

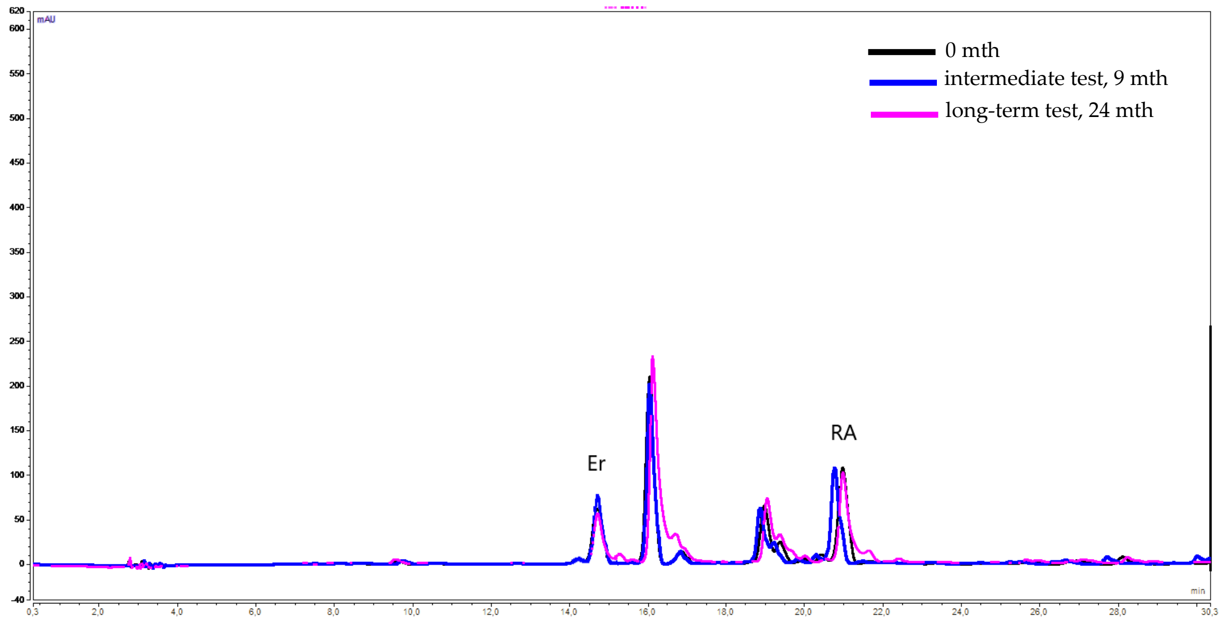

Figure S4. HPLC chromatograms of peppermint tincture b (PTb) in the three measurement points: 0, 9th (intermediate test), 24th month (long-term test) at 320 nm.

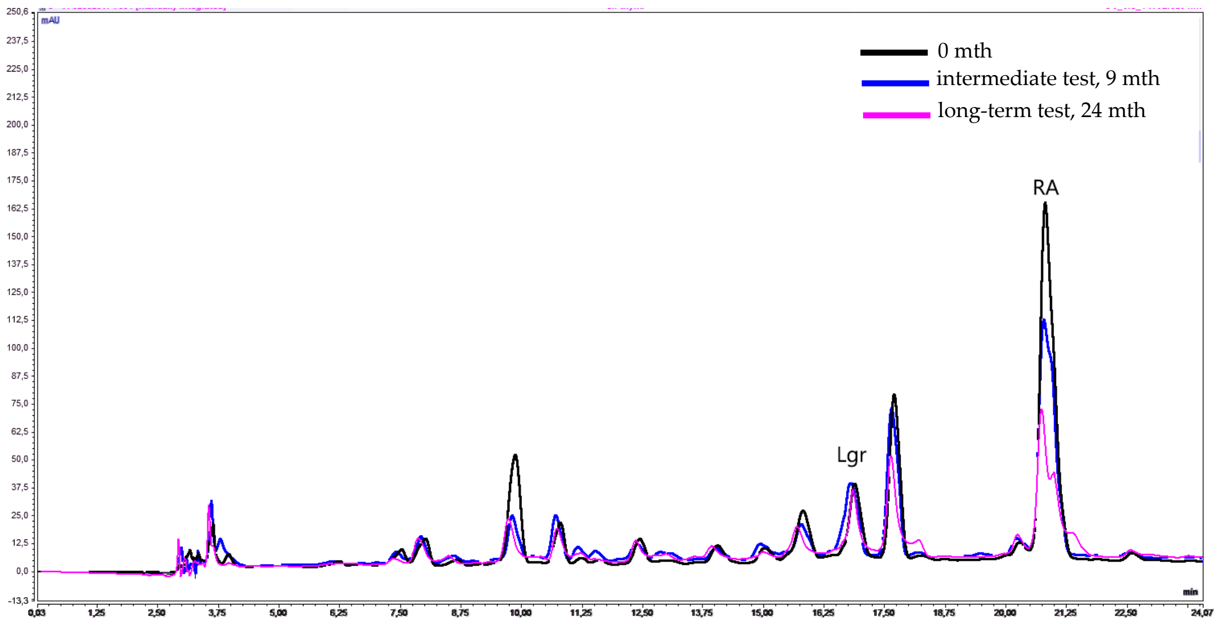

Figure S5. HPLC chromatograms of thyme syrup (TS) in the three measurement points: 0, 9th (intermediate test), 24th month (long-term test) at 320 nm.

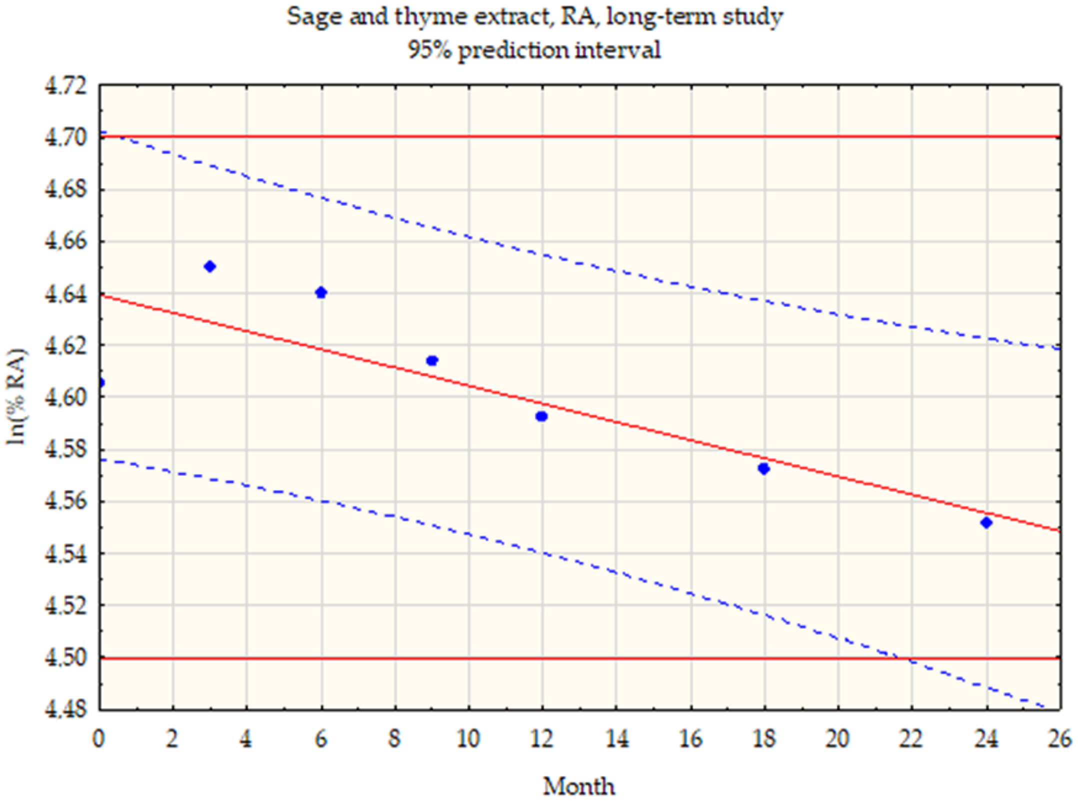

Figure S6. Sage and thyme liquid extract, rosmarinic acid (RA) content in the long-term test.

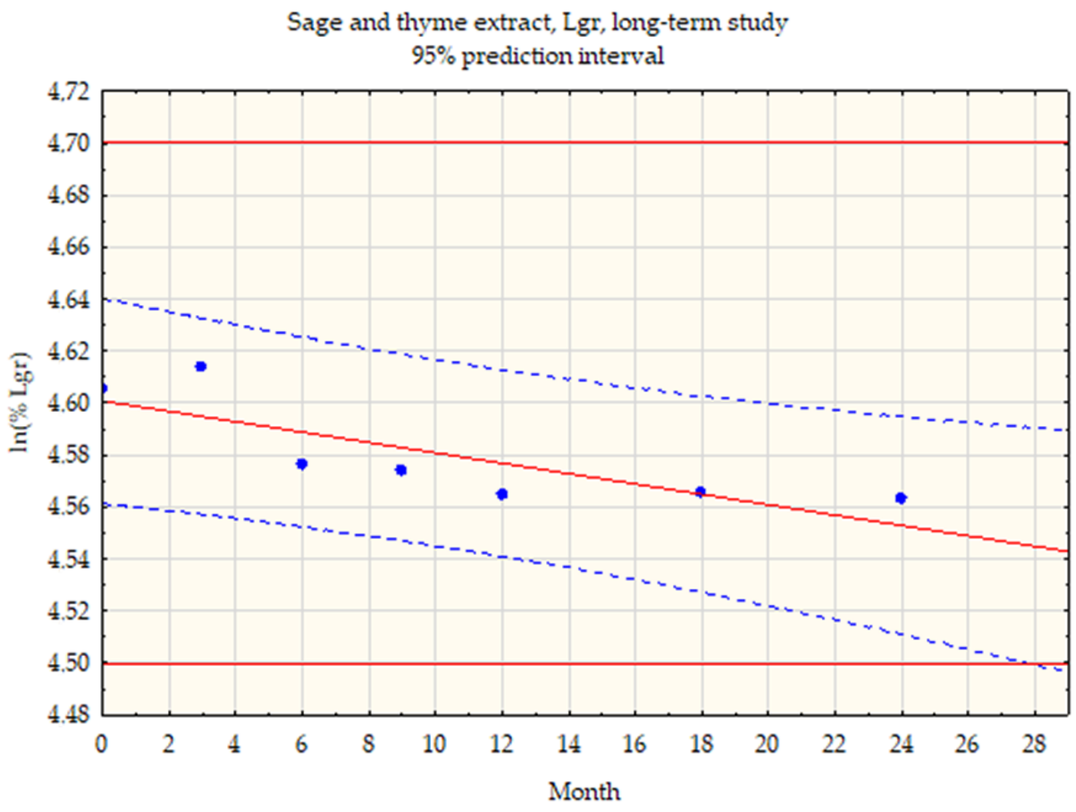

Figure S7. Sage and thyme liquid extract, luteolin-7-*O*-β-glucuronide (Lgr) content in the long-term test.

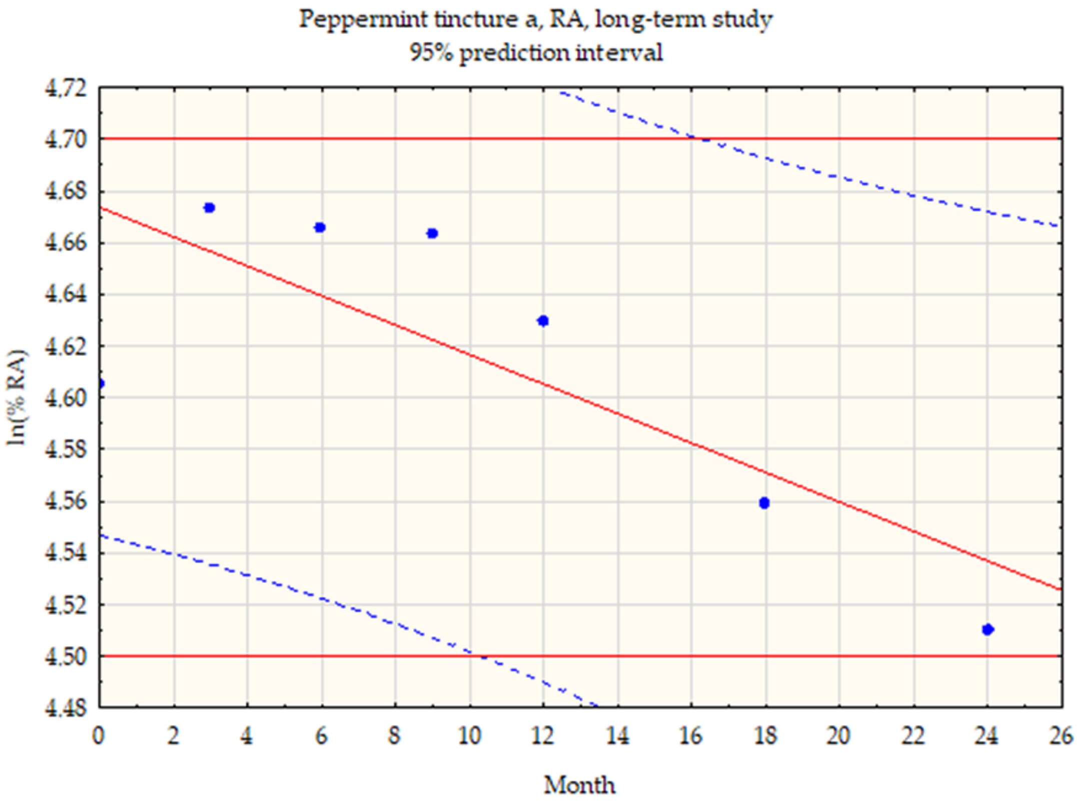

Figure S8. Peppermint tincture a, rosmarinic acid (RA) content in the long-term test.

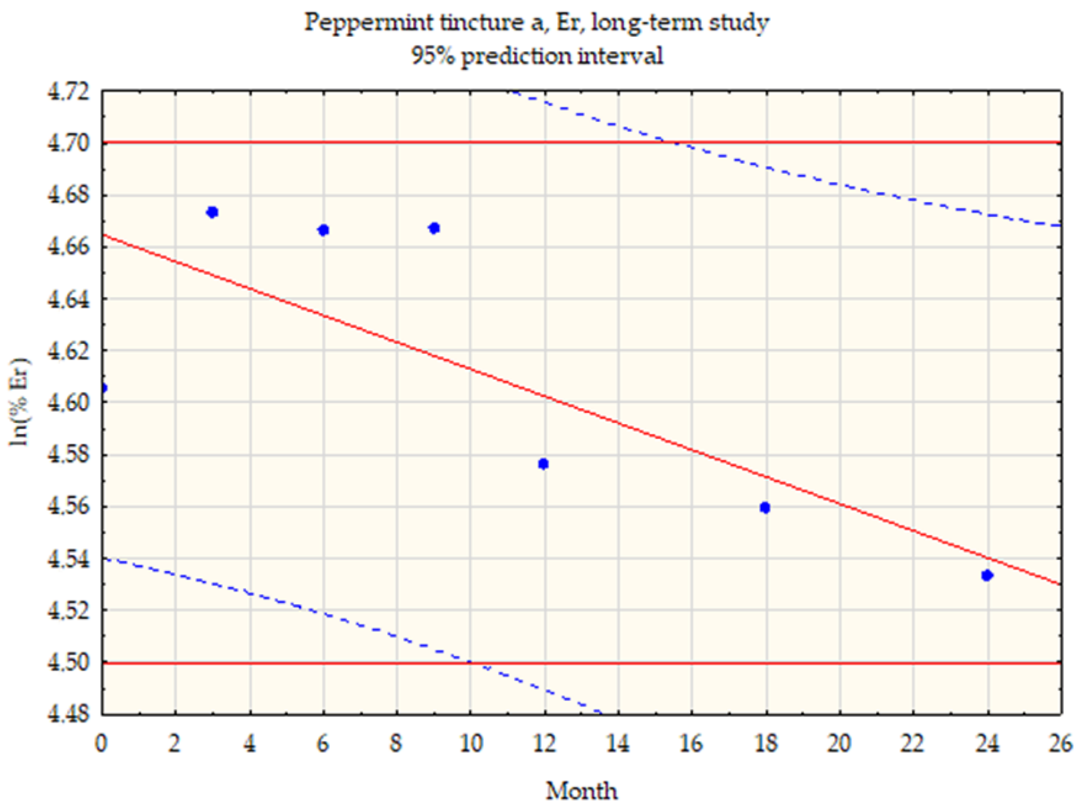

Figure S9. Peppermint tincture a, eriocitrin (Er) content in the long-term test.

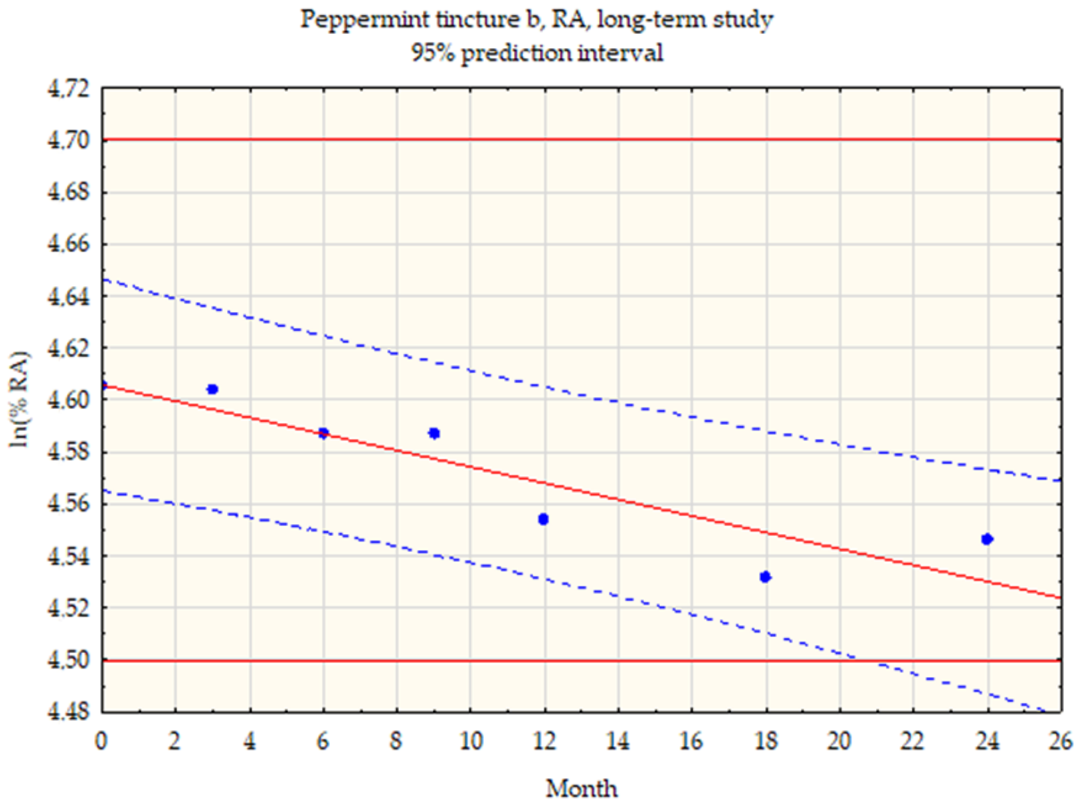

Figure S10. Peppermint tincture b, rosmarinic acid (RA) content in the long-term test.

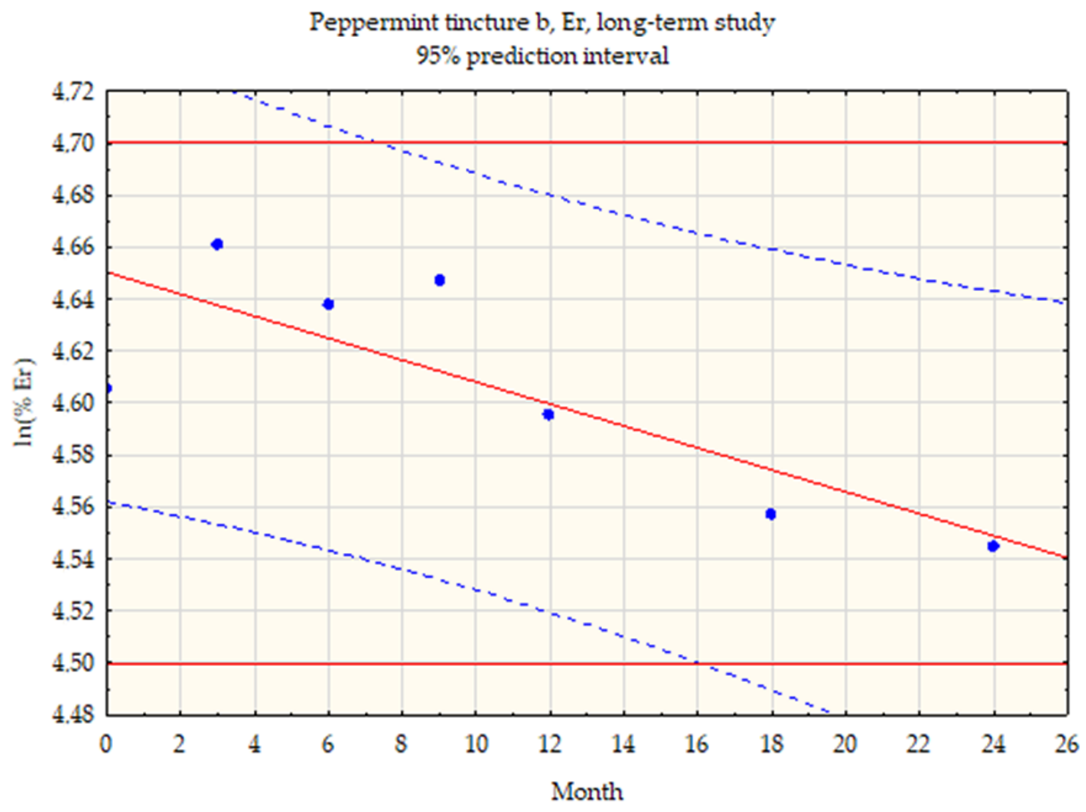

Figure S11. Peppermint tincture b, eriocitrin (Er) content in the long-term test.

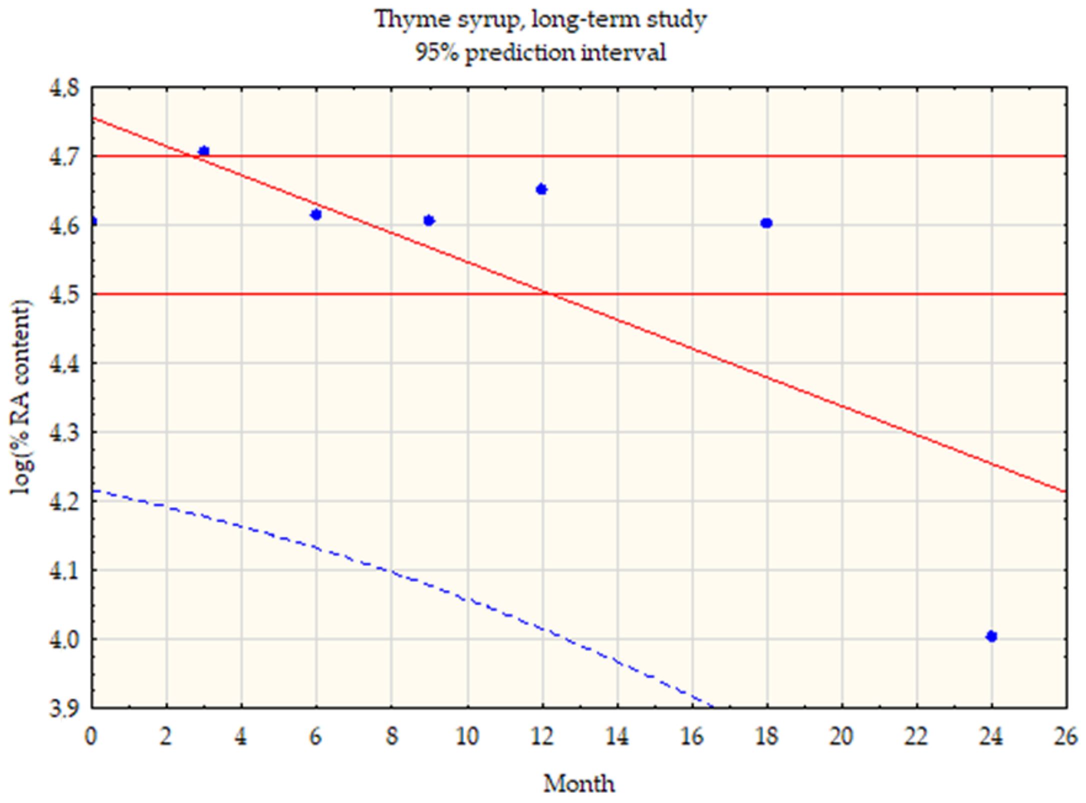

Figure S12. Thyme syrup, rosmarinic acid (RA) content in the long-term test.

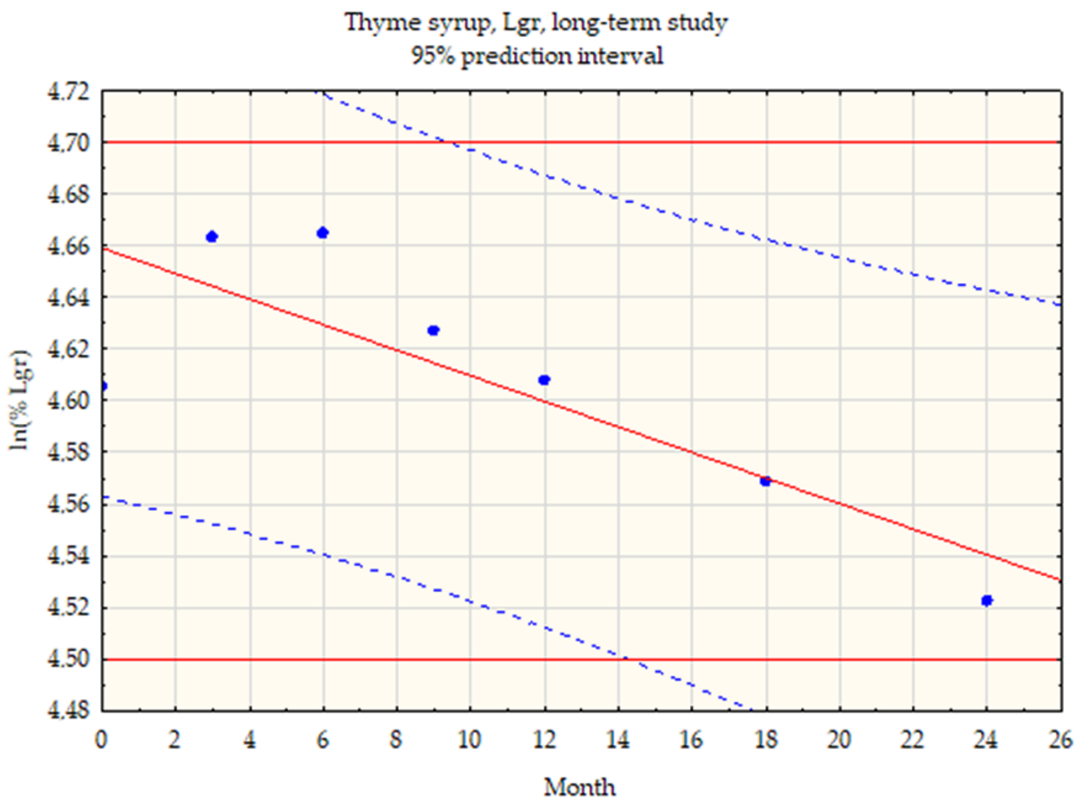

Figure S13. Thyme syrup, luteolin-7-*O*-β-glucuronide (Lgr) content in the long-term test.
